# Protonation Equilibria of Biologically Active Ligands in Mixed Aqueous Organic Solvents

**DOI:** 10.1155/2014/626719

**Published:** 2014-08-14

**Authors:** Ahmed A. El-Sherif, Mohamed M. Shoukry, Abeer T. Abd Elkarim, Mohammad H. Barakat

**Affiliations:** ^1^Chemistry Department, Faculty of Science, Cairo University, Giza, Egypt; ^2^Department of Chemistry, Faculty of Arts and Science, Northern Border University, Rafha, Saudi Arabia; ^3^Department of Chemistry, Faculty of Science, Islamic University, Al-Madinah, Saudi Arabia; ^4^Holding Company for Water and Waste Water, The Reference Laboratory for Drinking Water, Inorganic Laboratory, Cairo, Egypt

## Abstract

The review is mainly concerned with the protonation equilibria of biologically active ligands like amino acids, peptides, DNA constituents, and amino acid esters in nonaqueous media. Equilibrium concentrations of proton-ligand formation as a function of pH were investigated. Also, thermodynamics associated with protonation equilibria were also discussed.

## 1. Introduction

There is a growing interest in studying the properties of biologically active ligands like amino acids, peptides, DNA, and amino acid esters. Amino acids and peptides have special importance among the other chemical groups since they are the foundation stones of the living organisms. They are not only components of tissues, but also reactive organic compounds which are important regulators of biological processes. It is obvious that one has to know the physical properties of amino acids and peptides in order to explain the behavior and synthesis of proteins and enzymes in the organisms. The study of amino acids, peptides, or DNA units has been the subject of increasing research efforts, which have revealed the role of hydrogen ion at molecular level. These compounds are usually considered as good model systems to attain a better insight into the characteristics of naturally occurring copper metalloproteins [1]. The data related to the protonation constants of biologically active ligands in various media will be valuable in further understanding of their chemistry in biological systems. Also, analytical chemists are supposed to know the related constants of the species present in the medium to determine the accuracy and most suitable medium for their analysis.

The major reasons for the determination of protonation constants can be summarized as follows: one can calculate the pH and the ratio of different forms of a certain substance by the use of its protonation constants. Due to the fact that different forms of different substances have different UV spectra, by choosing a suitable pH value one can carry out spectrophotometric quantitative analyses. The choice of the pH value requires knowledge of protonation constants. Protonation constants are important in preparative chemistry as well. If the protonation constants of a certain substance are known, it is possible to isolate it with a maximum yield by finding the pH range where the compounds show minimum ionization. Protonation of a newly synthesized compound can also give supportive information about its structure. If theoretically calculated protonation constants are in good accordance with the experimental values, it is possible that the proposed structure could be correct. It is necessary that the protonation constants be known in order to prepare buffer solutions at different pH values [2]. There are various techniques such as conductometry, spectrophotometry, and potentiometry [3–5] that are used in the determination of protonation constants. In addition, for the calculations of stability constants of the complex formation of biologically active ligands with metal ions, their protonation constants are used [6]. Knowledge of the equilibrium constants of some compounds is necessary for the calculation of the concentration of each ionized species at any pH, which is important for the complete understanding of the physicochemical behavior of such molecules [6].

Mostly, physical constants of bioligands are studied in aqueous media. Despite this, little is known about the chemistry of biomolecules in the mixtures of organic solvents and water, in regard to either protonation constants or synthetic applications. One reason for this dearth of knowledge is that* in vivo* reactions take place in aqueous media, so that interest in bioligands properties in aqueous solution has predominated. However, in the literature it has been shown that water is not an ideal model for* in vivo* reactions. In enzymes, membranes, and other biologically important media, the p*K*_a_ values are far different from those in water, as these media tend to be lipophilic rather than hydrophilic [7, 8]. Studies in media other than water should provide some understanding of the chemistry of bioligands in living systems. With this in mind and in continuation of our research program directed to study the solution equilibria of biologically active compounds [9–15], the present review discusses the valuable information existing in the literature for some bioactive ligands like amino acids, amino acid esters, peptides, and DNA constituents in nonaqueous media.

## 2. Determination of Protonation Constants of Biologically Active Ligands

The determination of equilibrium constants is an important process for many branches of chemistry [16]. Developments in the field of computation of equilibrium constants from experimental data were reviewed by Legget [17] and Meloum et al. [18]. Since that time, many more programs have been published, mainly so as to be able to use microcomputers for the computations. The most commonly used programs for solution equilibrium constant determination are PKPOT [19], PKAS [20], BEST [21], MINIQUAD [22], MINIQUAD-75 [23], SUPERQUAD [24], PSEQUAD [25], and HYPERQUAD [26]. All of these programs use least-square refinements to reduce the differences between calculated and experimental data to get the best model that gives the best fit. The sum of square of residuals between experimental and calculated values is normally very small; it is typically between 10^−6^ and 10^−9^.

## 3. Protonation Equilibria in Nonaqueous Solutions

Vast data are available on the protonation and stability constants of the bioligands in aqueous solutions [27–40]. But the knowledge which may be obtained from mixed solvents could complement the vast amount of data collected from studies on the chemistry of bioligands in aqueous media. Water-organic solvent mixtures have attracted much interest because of their frequent use and wide field of applications. These solvent mixtures are also used as reaction media for a variety of organic and analytical processes such as synthesis, titrations, or liquid chromatographic separations. Solvent molecules act as proton acceptors and therefore the solvent plays a crucial role in chemical and biochemical reactions. The acid-base behavior of substances in these mixtures is of considerable interest and importance. Data related to protonation constants will be valuable in the further understanding of biological systems. Hence, studies in media other than water should provide some understanding of the chemistry of bioligands in living systems.

### 3.1. Autoprotolysis Constants (*K*_ap_)

The autoprotolysis constant (*K*_ap_) of a solvent is an important parameter in understanding acid-base equilibria in mixed solvents. It also determines the extreme limits of acidity and basicity in a given solvent medium. The dielectric constant and the intrinsic acidic and basic strength of the solvent will influence the self-ionization process in these media. The recent IUPAC document on the criteria for standardization of pH measurements in organic solvent and water-organic solvent mixtures underlines the importance of knowing the autoprotolysis constant (in fact, −log⁡_10_⁡*K*_ap_ = normal scale length of pH) pertaining to each solvent considered, which can be achieved by appropriate thermodynamic methods [41]. Various methods have been used to obtain values of the autoprotolysis constants in aqueous organic solvent mixtures by potentiometry [42]. These experimental methods are difficult, and those methods that make use of the hydrogen electrode can only be applied to systems in which there is no complication due to reduction at the hydrogen electrode. Furthermore, Rosés et al. [43, 44] derived equations to calculate autoprotolysis constants of aqueous organic solvent mixtures and they expressed that all of the autoprotolysis processes are determined by the polarity and hydrogen bounding capability of solvents. Kiliç and Aslan [45] derived a convenient and rapid potentiometric technique using a combined glass-pH electrode for the determination of autoprotolysis constants in a variety of aqueous organic mixed solvents. For different compositions of organic solvent-water mixtures, reproducible values of autoprotolysis constants were calculated from several separate series of [H^+^] and [OH^−^] measurements as given in Tables 1 and 2 [45–51].

### 3.2. Protonation Equilibria in Mixed Ethanol-Water Medium

Among the organic solvents, ethanol is closest to water in structure and properties and therefore the behaviour of dissociation or protonation in ethanol is similar to that of aqueous solution. The water-ethanol mixture is a very interesting binary mixture. One reason is that ethanol can dissolve a majority of organic acids and bases more effectively than water. However, it has been suggested that solvents such as water-ethanol mixtures provide a better model for* in vivo* reactions [52, 53] because the mixtures simultaneously show a low polar character and partially aqueous contents, as do all biological systems. In addition, ethanol-water mixtures are a suitable solvent for the determination of equilibrium constants [53–56]. In addition, there has been increasing interest during the past few years in the properties of mixed-solvent systems such as polar aprotic solvents and water. Water and organic solvents such as methanol, ethanol, acetone, and acetonitrile are completely miscible. Their mixtures are macroscopically homogeneous, but it has been reported that the water and organic solvent molecules are not homogeneously dispersed microscopically, because of the hydrogen-bonding network formation and hydrophobic interactions [46]. Consequently, the molecular composition of the solvation layer around a solute molecule is not the same as that of the bulk mixing ratio of water and organic solvent. The hydrophobic and hydrophilic properties of a solute may be reflected by preferential solvation in such mixed solvents. Therefore, the influence which the solvent exerts on the log⁡_10_⁡*K* values depends upon the extent and nature of the solute-solvent interaction, which involves species participating in the acid-base equilibrium. Also, analytical chemists are supposed to know the related constants of the species present in the medium to determine the accuracy and most suitable medium for their analysis. Protonation constants of some *α*-amino acids at different water-ethanol mixtures are given in Tables 3–7 [45, 55, 57–63].

#### 3.2.1. Protonation Equilibria of Amino Acids in Mixed Water-Ethanol Medium

**Figure 1:**
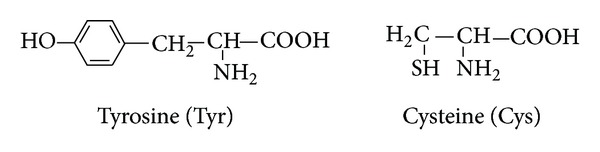


*(1) Protonation Equilibria of L-Cysteine and L-Tyrosine in Mixed Water-Ethanol Medium (Figure 1).* The protonation constants of L-cysteine and L-tyrosine given in Table 3 correspond to the following equilibria:

(1) $$\begin{array}{c} {\text{L}}^{2-}+{\text{H}}^{+}⇌{\text{HL}}^{-}\;\;K_{1}=\frac{\left[\text{H}{\text{L}}^{-}\right]}{\left[{\text{L}}^{2-}\right]\left[{\text{H}}^{+}\right]}\\ \text{H}{\text{L}}^{-}+{\text{H}}^{+}⇌{\text{H}}_{2}\text{L}\;\;K_{2}=\frac{\left[{\text{H}}_{2}\text{L}\right]}{\left[\text{H}{\text{L}}^{-}\right]\left[{\text{H}}^{+}\right]}\\ {\text{H}}_{2}\text{L}+{\text{H}}^{+}⇌{\text{H}}_{3}{\text{L}}^{+}\;\;K_{3}=\frac{\left[{\text{H}}_{3}{\text{L}}^{+}\right]}{\left[{\text{H}}_{2}\text{L}\right]\left[{\text{H}}^{+}\right]} \end{array}$$

The log⁡_10_⁡*K*_1_ values are related to the attachment of H^+^ to the phenolic oxygen in L-tyrosine and attachment of H^+^ to the –NH_2_ group in L-cysteine. The log⁡_10_⁡*K*_2_ value corresponds to the attachment of a proton to the –NH_2_ and sulfur thiol groups in L-tyrosine and L-cysteine, respectively. The log⁡_10_⁡*K*_3_ values are the smallest and are thought to correspond to the protonation of carboxyl groups. The decrease in the log⁡_10_⁡*K*_2_ value with increasing ethanol concentration can be explained by the fact that the dipolar ionic form is more strongly solvated than the anionic form in aqueous media.

**Figure 2:**
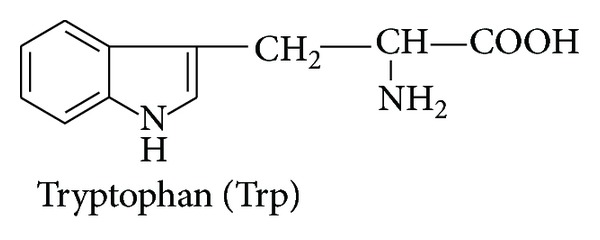


*(2) Protonation Equilibria of L-Tryptophane in Mixed Water-Ethanol Medium (Figure 2).* Two protonation equilibria were found for L-tryptophan. The first protonation constant was attributed to the protonation of –NH_2_ and the second one was related to protonation of carboxyl group. No protonation constants were found related to the protonation of the nitrogen atom of the indole ring of this amino acid. The decrease in log⁡_10_⁡*K*_1_ values of L-tryptophane was observed with the increase of ethanol concentration. This can be explained by better solvation of the dipolar ionic form.

**Figure 3:**
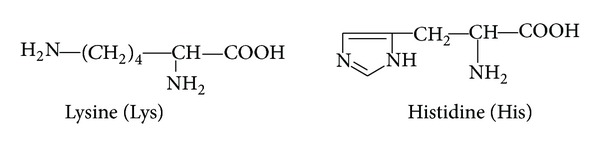


*(3) Protonation Equilibria of L-Lysine and Histidine in Mixed Water-Ethanol Medium (Figure 3).* L-lysine contains –NH_2_ group in the side chain. Due to the fact that this amino group is far from the carboxyl groups, resulting in a lesser degree of inductive effect, it can be said that log⁡_10_⁡*K*_1_ is related to the protonation of this amino group. The second and third protonation equilibria belong to the *α*-amino and carboxyl groups, respectively. For L-histidine, the log⁡_10_⁡*K*_1_ and log⁡_10_⁡*K*_2_ values are related to the protonation of –NH_2_ and the nitrogen in imidazole groups, respectively. The third constant (log⁡_10_⁡*K*_3_) belongs to the protonation of the carboxylate group.

The log⁡_10_⁡*K*_1_ and log⁡_10_⁡*K*_2_ values of L-lysine decrease with increasing ethanol concentration in the solvent mixture. Although, in the first equilibrium involving *K*_1_, HL seems to be an uncharged species, it belongs to the dipolar form of amino acid. If we consider the solvation of these species, the fact that both *K*_1_ and *K*_2_ decrease with increasing ethanol concentration seems highly reasonable. The fact that the log⁡_10_⁡*K*_1_ value of L-histidine decreases with increasing ethanol concentration can be explained by the fact that dipolar HL is better solvated in water than anionic form (L^−^). The reason for the decrease in log⁡_10_⁡*K*_2_ is that solvation of the multicharged species formed by the attachment of a proton to the imidazole ring is very limited in ethanol.

*(4) Protonation Equilibria of Some *α*-Amino Acids in Mixed Water-Ethanol Medium.* The numerical log⁡_10_⁡*K*_1_ and log⁡_10_⁡*K*_2_ values of eight *α*-amino acids (glycine, L-alanine, L-valine, L-leucine, L-isoleucine, L-phenylalanine, L-serine, and L-methionine) determined in water-ethanol mixtures are given in Table 4. When the change of log⁡_10_⁡*K*_1_ given in Table 4 with the solvent composition is examined, it is observed that these values decrease with the increase in the mole fraction of ethanol. This can be explained by the structural changes in amino acids as the medium changed toward the ethanol-rich direction. The neutral form amino acid HL is subjected to higher solvation compared with L^−^ forms in ethanol rich media. In water-rich media, however, the reverse behavior will be the case. This shows that log⁡_10_⁡*K*_1_ values are expected to decrease with an increase in ethanol ratio. Therefore, one can conclude that the dipolar form of amino acids, HL^±^, dominates in these media. It is the only way to explain the decrease in log⁡_10_⁡*K*_1_ as the amount of ethanol in the media is increased since L^−^ is expected to solvate more than HL^±^ in media rich in ethanol.

The fact that log⁡_10_⁡*K*_2_ values are observed to increase as the mole fraction of ethanol is increased in all media investigated supports that dipolar ion is predominant in water-ethanol mixtures as well as in water. This is due to the fact that ethanol solvated H_2_L better than HL^±^. If the neutral form were the predominant species in water-ethanol mixture, log⁡_10_⁡*K*_2_ values would decrease with increasing amount of ethanol since ethanol solvated HL better than H_2_L^+^ [64, 65].

#### 3.2.2. Protonation Equilibria of Dipeptides in Mixed Water-Ethanol Medium

**Figure 4:**
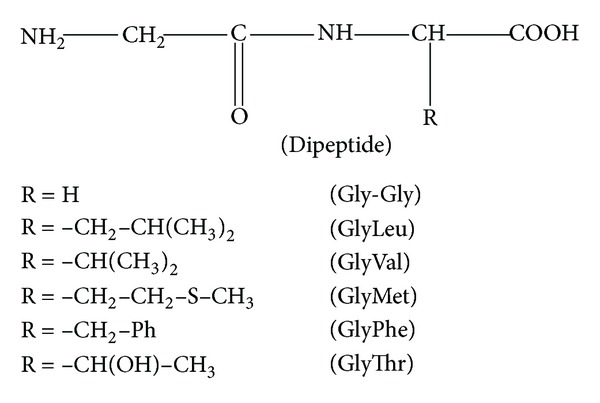


Two protonation equilibria were found for all glycine-dipeptides (Figure 4) mentioned in Table 5.

The first protonation constant was attributed to the protonation of amino group and the second one was related to the protonation of carboxylate group:

(2) $$\begin{array}{c} {\text{L}}^{-}+{\text{H}}^{+}⇌\text{HL}\;\;K_{1}=\frac{\left[\text{HL}\right]}{\left[{\text{L}}^{-}\right]\left[{\text{H}}^{+}\right]}\\ \text{HL}+{\text{H}}^{+}⇌{\text{H}}_{2}{\text{L}}^{+}\;\;K_{2}=\frac{\left[{\text{H}}_{2}{\text{L}}^{+}\right]}{\left[\text{HL}\right]\left[{\text{H}}^{+}\right]} \end{array}$$

where L is dipeptide monoanion; HL is total zwitterion and neutral forms of dipeptide.

Chattopadhyay and Lahiri [66] examined the effect of a change of solvent composition on BH^+^ ionization and the related Gibbs transfer energies in mixed solvent. They emphasized that the electrostatic charge effects due to changes in the dielectric constants with changes in solvent composition are of minor importance in explaining solvent effects, and that solute-solvent interactions have greater significance in interpretation of solvent effects.

These protonation constants have been considered in some detail to gain more information about the effect of solvent composition on the corresponding equilibria. For this purpose, by examining the protonation equilibria of dipeptides with solvent composition, it is generally observed that log⁡_10_⁡*K*_1_ values decrease with the increase in the mole fraction of ethanol, whereas log⁡_10_⁡*K*_2_ values increase as the composition of ethanol increases in water-ethanol mixture. The decrease in log⁡_10_⁡*K*_1_ values and increase in log⁡_10_⁡*K*_2_ values of all dipeptides with the increasing ethanol concentration were the same as for the corresponding free amino acids [48].

The fact that the variation of log⁡_10_⁡*K*_1_ and log⁡_10_⁡*K*_2_ of dipeptides with solvent composition is similar to those of the corresponding free amino acids and can be concluded from the domination of the zwitterionic form of dipeptides in water-ethanol mixtures. In water-rich media, however, the reverse behavior will be the case. This shows that log⁡_10_⁡*K*_1_ values are expected to increase with an increase in the ethanol ratio. This is the only way which explains the decrease in log⁡_10_⁡*K*_1_ as the amount of ethanol in the media is increased because L^−^ is expected to solvate more than the zwitterionic form of dipeptides in the media rich in ethanol. The fact that log⁡_10_⁡*K*_2_ values are observed to increase as the mole fraction of ethanol is increased in all media investigated supports that dipolar ion is predominant in water-ethanol mixtures as well as in water. This is due to the fact that ethanol solvated H_2_L better than zwitterionic form. If the neutral form were the predominant species in water-ethanol mixture, log⁡_10_⁡*K*_2_ values would decrease with increasing amount of ethanol because ethanol solvated HL better than H_2_L^+^. Thus, we can say that the log⁡_10_⁡*K*_2_ values for these dipeptides increase as the mole fraction of ethanol increases.

In order to compare the log⁡_10_⁡*K*_1_ and log⁡_10_⁡*K*_2_ dipeptides with the log⁡_10_⁡*K*_1_ value of glycine and log⁡_10_⁡*K*_2_ value of the other amino acid forming the dipeptide the protonation constants of the corresponding amino acids are given in Table 5. According to the data given in Table 5, the log⁡_10_⁡*K*_2_ values of amino acids increase and the log⁡_10_⁡*K*_1_ values of glycine decrease due to peptide formation. For instance log⁡_10_⁡*K*_1_ and log⁡_10_⁡*K*_2_ values for glycine in 60% water-40% ethanol medium were 9.39 and 2.89 [55, 67], respectively, while same values were found as 6.92 and 3.54 for Gly-Gly. Similarly log⁡_10_⁡*K*_1_ for glycine was 9.20 [55] and log⁡_10_⁡*K*_2_ for phe was 3.05 [57] in 40% water-60% ethanol medium while these values were observed to be 6.98 and 3.91 for GlyPhe in the same medium. This may be attributed to the fact that the electron density of the amino group in dipeptide is lower than that of glycine, and the electron density of the carboxylate group is higher than that of the corresponding amino acid due to the formation of a peptide bond.

**Figure 5:**
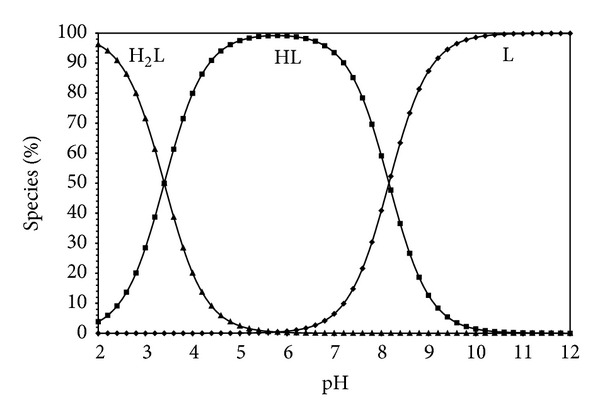
Species distribution diagram (25°C, *I* = 0.1 M NaCl) for Gly-Gly system as a function of pH in water (L = 1.5 × 10^−3^ M).

**Figure 6:**
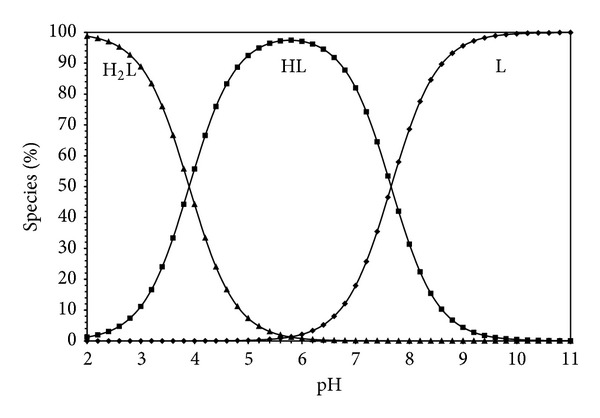
Species distribution diagram (25°C, *I* = 0.1 M NaCl) for Gly-Gly system as a function of pH in 20% ethanol-80% water (L = 1.5 × 10^−3^ M).

**Figure 7:**
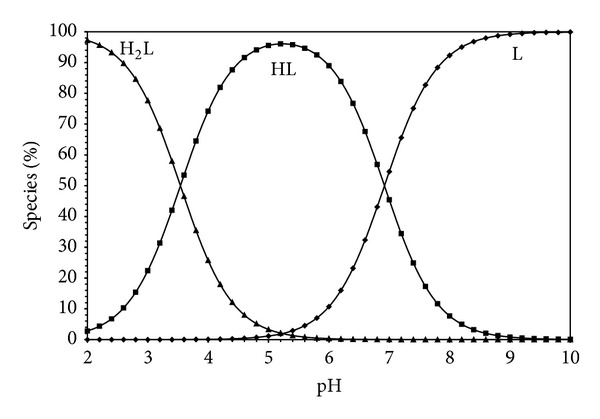
Species distribution diagram (25°C, *I* = 0.1 M NaCl) for Gly-Gly system as a function of pH in 40% ethanol-60% water (L = 1.5 × 10^−3^ M).

**Figure 8:**
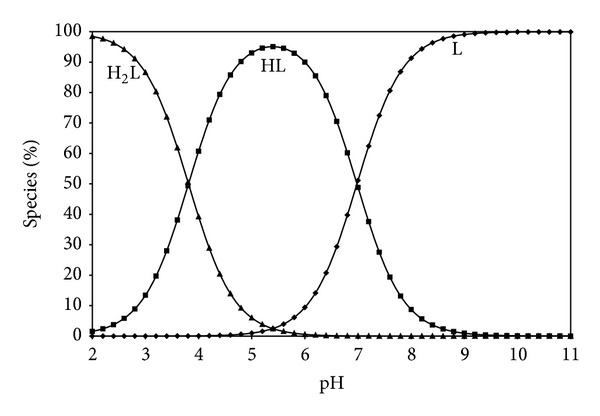
Species distribution diagram (25°C, *I* = 0.1 M NaCl) for Gly-Gly system as a function of pH in 60% ethanol-40% water (L = 1.5 × 10^−3^ M).

Estimation of equilibrium concentrations of proton-ligand formation as a function of pH provides a useful picture of proton-ligand binding in media [16]. Figures 5, 6, 7, and 8 show the change of different species of dipeptides with pH. It is seen from these figures that the most predominant species is HL (total concentration of the zwitterion and the neutral form) between pH 5 and 7 in all ethanol-water media.

#### 3.2.3. Protonation Equilibria of Amino Acid Esters in Ethanol-Water Medium

The changes of the protonation constants of methyl and ethyl esters of L-tyrosine, L-tryptophane, L-cysteine, L-lysine, and methyl ester of L-histidine with increasing ethanol percentage are given in Table 6. It can be seen that log⁡_10_⁡*K*_1_ values of L-tyrosine, L-tryptophane, and L-cysteine esters increase with increasing percentage of ethanol. On the other hand, the log⁡_10_⁡*K*_2_ values show a decrease with increasing ethanol concentration. The log⁡_10_⁡*K*_1_ and log⁡_10_⁡*K*_2_ values of L-lysine and L-histidine esters both decrease with increasing ethanol concentration in the solvent mixture.

The protonation constants of methyl and ethyl esters of L-cysteine, L-tyrosine, and L-tryptophane are given in Table 6. The log⁡_10_⁡*K*_1_ values belong to equilibria related to the attachment of H^+^ to the sulfur atom in the thiol group of cysteine esters, to the nitrogen atom in the indole ring of tryptophane, and to the phenolic oxygen group in tyrosine esters. The log⁡_10_⁡*K*_2_ values are related to the protonation of –NH_2_ groups in the esters. The increase in the ethanol concentration decreases the dielectric constant of the medium. The amino acid ester (HL) is subjected to stronger solvation compared with the (L^−^) and (H_2_L^+^) forms in ethanol-rich media. In water-rich media, however, the reverse behavior will be the case. This shows that log⁡_10_⁡*K*_1_ values are expected to increase and log⁡_10_⁡*K*_2_ values to decrease with an increase in the ethanol ratio.

The log⁡_10_⁡*K*_1_ and log⁡_10_⁡*K*_2_ values of L-lysine esters are related to the protonation of amino groups in side chain and *α*-amino groups, respectively. For L-histidine esters, these values correspond to the protonation of nitrogen in the imidazole and *α*-amino group in L-histidine. Contrary to the other esters investigated both log⁡_10_⁡*K*_1_ and log⁡_10_⁡*K*_2_ values of lysine methyl and ethyl esters and histidine methyl ester decrease with an increase in the ratio of ethanol in the solvent mixture. Since H_2_L^2+^ is better solvated than HL^+^ and the latter is better solvated than L-molecular species in water-rich media, therefore, results obtained for these esters can be explained by the specific solvation effect.

The stoichiometric protonation constants for t-butyl esters and benzyl esters of *α*-amino acids determined in ethanol-water mixtures (20–80 volume % ethanol) are given in Table 7. These values are related to this equilibrium reaction L + H^+^⇌LH^+^, where L and *LH*⁡^+^ show *α*-amino acid esters and their protonated species, respectively. The protonation constants given in Table 7 are considered in more detail in order to gain more information about the effect of solvent composition and specific effects of substituents on the basicities of the esters in solvent mixtures. The numerical log⁡_10_⁡*K* values for t-butyl esters of *α*-amino acids determined in ethanol-water mixtures decrease with increasing ethanol content in the solvent mixture. It is observed that a nearly linear relationship exists between the aforementioned protonation constants and the mole fraction of ethanol from 0.0331 to 0.4183 for all *α*-amino acid esters investigated. However, log⁡⁡*β* values at a mole fraction of ethanol of 0.4183 are slightly higher than those expected from the linear trend. The linear variation for all *α*-amino acid esters that are given in Table 7 is very similar to that found for ammonia aliphatic alkyl amines, pyridine, and salicylideneanilines [68, 69]. The dissociation constants of charged acids in water-ethanol mixtures vary with solvent composition in a manner that is not completely understood. It is suggested that electrostatic charging effects resulting from the change in dielectric constant with solvent effects and the solute-solvent interactions have greater significance in the interpretation of solvent effects. Thus, results obtained for *α*-amino acid esters in water-ethanol medium can be explained by specific solvation effects. Since ethanol would solvate L better than *LH*⁡^+^, the log⁡⁡*β* values, which are related to the formation of *LH*⁡^+^, would decrease upon addition of ethanol. The derivations of linearity in 80% ethanol may result from the preferential solvation of solute by one of the components of the solvent mixture that could change the effective dielectric constant value in the cybotactic region [70]. Furthermore, another factor which explains the increase in the log⁡⁡*β* values of all *α*-amino acid esters in ethanol-rich regions is the differences in the solvent stabilization of the ionic species (H^+^ and *LH*⁡^+^), brought about by changing the percentage of ethanol [5, 71]. Using the protonation constants obtained in this paper, the effects of the type of ester groups on the basicity of the amine groups of *α*-amino acid esters have been discussed. The most important factor that affects the basicity and therefore the protonation constant of a compound is the structural effect. Table 7 showed that the basicity of t-butyl esters of glycine, L-alanine, and L-valine is higher than that of the corresponding benzyl esters of the same amino acids in 20%–60% ethanol-water mixtures. This effect can be explained by taking the electronic effect of the t-butyl and benzyl groups investigated into account.

### 3.3. Protonation Equilibria in Mixed Water-Dioxane Medium

Simple heterocyclic compounds, such as 1,4-dioxane, in pesticides are very important to living organisms and environment. Also, it is well established that the “effective” or “equivalent solution” dielectric constants in protein [72, 73] or active site cavities of enzymes [74] are small compared to that in bulk water. Estimates for the dielectric constants in such locations range from 30 to 70 [73, 75]. Hence by using aqueous solutions containing ~10–50% dioxane, one may expect to simulate to some degree the situation in active site cavities [76] and hence to extrapolate the data to physiological conditions. Mixed solvents such as water-dioxane mixtures provide a better model for* in vivo* reactions because the mixtures simultaneously show a lesser polar character and are partially aqueous contents, as do all biological systems [2]. On the other hand, water-1,4-dioxane mixtures are a very interesting binary solvent system. These mixtures are a favorite mixed solvent system in which to study association and mobilities of ions, because the dielectric constant can be varied over a large range. Changes in protonation constants upon addition of 1,4-dioxane to aqueous solutions are due to increasing ion-ion interactions resulting from the decreasing dielectric constant and from changes in solvent-ion and solvent-solvent interactions [77–79]. Also, the protonation constant of amino acid esters will be helpful in the determination of the microscopic equilibrium constants of the corresponding amino acids. Protonation constants of some *α*-amino acids at different water-dioxane mixtures are given in Tables 8 and 9 [49, 67].

#### 3.3.1. Protonation Equilibria of *α*-Amino Acids in Mixed Dioxane-Water Medium

**Figure 9:**
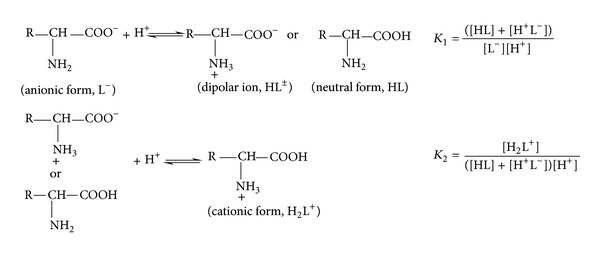


The stoichiometric protonation constants of eight *α*-amino acids determined in various water-dioxane mixtures are listed in Table 8. The log⁡_10_⁡*K*_1_ and log⁡_10_⁡*K*_2_ values given in Table 8 refer to the protonation equilibria shown in Figure 9, respectively.

Many studies have shown that the equilibrium constant is linearly related to the fraction of organic solvent [80–83]. The results obtained in this study confirm this observation. Regarding the variation of log⁡_10_⁡*K*_1_ values with the solvent composition, one can postulate that the zwitterionic to neutral form ratio decreases as the dioxane content increases. This can be inferred from considerations on specific solute-solvent interactions and structural changes in amino acids from water to dioxane-water media. In the related equilibrium, the HL form would be more solvated than the L^−^ and the HL^±^ forms in solvents containing a higher concentration of dioxane, whereas the opposite forms would hold for a solvent rich in water. If the zwitterionic to neutral form ratio (*K*_*Z*_) was unaltered with increasing percentage of dioxane, the log⁡_10_⁡*K*_1_ values would be expected to decrease, which is not observed in the present case. If the neutral form became the predominant species when dioxane content is increased, the log⁡_10_⁡*K*_1_ values would increase as in the case of a neutral monobasic acid, whereas they are not altered [84]. Thus, even with the limitations, we can conclude that the *K*_*Z*_ values for all amino acids studied decrease as the concentration of dioxane increases. The fact that the log⁡_10_⁡*K*_2_ values increase with increasing the dioxane content for all the amino acids suggests that the zwitterion is the predominant species in dioxane-water mixtures as well as in water. This is based on the assumption that dioxane solvates H_2_L^+^ better than HL^±^. If the neutral form was the predominant species in water-dioxane mixtures, the log⁡_10_⁡*K*_2_ values would decrease on addition of dioxane since dioxane would solvate HL better than H_2_L^+^.

#### 3.3.2. Protonation Equilibria of Amino Acid Esters in Mixed Water-Dioxane Medium

The stoichiometric protonation constants of the *α*-amino acid esters and the effect of the solvents upon the values of these protonation constants of *α*-amino acid esters were discussed in water-1,4-dioxane mixtures and the results are tabulated in Table 9. These values are related to the protonation of –NH_2_ group of the esters, as follows:

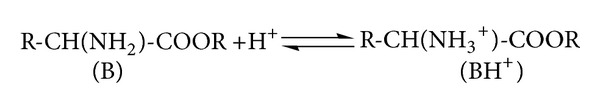
(3)
where B and BH^+^ denote the *α*-amino acid esters and their protonated species, respectively. The protonation constants given in Table 9 were considered in more detail in order to gain more information about the effect of solvent composition and specific effects of substituents on the basicities of the esters in solvent mixtures. When it comes to the variation of log⁡⁡*β* with the solvent composition, it can be observed that these constants decrease as the concentration of 1,4-dioxane increases in going from water to 60% in dioxane-water mixtures. It is also observed that a nearly linear relationship exists between the protonation constants and the mole fraction of 1,4-dioxane between 0.05 and 0.24 for all *α*-amino acid esters investigated. Many studies have shown that the equilibrium constant is linearly related to the fraction of organic solvent [80–83]. Although the effect of solvent composition upon the protonation constants of various compounds has been the subject of extensive investigation, the variation of charged acid-base equilibrium constants with solvent composition is yet to be fully elucidated. The solvation of a solute in a mixed solvent is much more complicated than solvation in a single pure solvent, and in the literature there are several different explanations. In organic solvent-water mixtures, acid-base equilibria can be estimated from two effects: an electrostatic one that can be explained by the Barron equation and a nonelectrostatic one that includes specific solute-solvent interaction [85]. Bates [71] and Chattopadhyay and Lahiri [66] have examined the effect of changing solvent composition on ionization of BH^+^ and the related Gibbs energies of transfer in mixed solvents, and they found that the dielectric constant alone cannot serve for a quantitative explanation for the solvent effect [66, 71]. However, the solvent mixtures are characterized by dramatic changes in the physical constants of the solvent (i.e., their melting and boiling point, dielectric constants, etc.) and macroscopic solvent parameters (mole fraction of cosolvent, etc.), upon changes of their compositions that influence many solute properties of these mixtures. Also, according to Takamuku et al. [86], the 1,4-dioxane-water mixtures can be divided into three mole fraction ranges, that is, 0.3 ≤ *X*_dioxane_ ≤ 0.9, *X*_dioxane_, 0.1 ≤ *X*_dioxane_ ≤ 0.2, and 0 ≤ *X*_dioxane_ ≤ 0.07. When 0.3 ≤ *X*_dioxane_ ≤ 0.9, the structure observed for pure dioxane obviously remains the same as it is mixed with 0.1 ≤ *X*_dioxane_ ≤ 0.2, small binary clusters consisting of one or two 1,4-dioxane molecules and several water molecules are formed by hydrogen bonding. When 0 ≤ *X*_dioxane_ ≤ 0.07, first-, second-, and third-neighbor O ⋯ O interactions are observed due to the hydrogen-bonded network of water. This suggests that the addition of a small amount of 1,4-dioxane breaks down the hydrogen-bonded network of water. In dioxane-rich mixtures the protonated base (BH^+^) is mostly solvated by the less basic dioxane molecules, whereas the proton is more solvated in the water-dioxane mixtures than in water and dioxane, because the water-dioxane mixture is more basic than either pure water or dioxane. Also, the molecular composition of the solvation layer around a solute molecule is not the same as that of the bulk mixture ratio of water to organic solvent. The hydrophobic and hydrophilic properties of a solute may be reflected in preferential solvation in such mixed solvents. Therefore, the influence that the solvent exerts on the p*K* values depends upon the extent and nature of the solute-solvent interactions, which involves species participating in the acid-base equilibrium [72]. From the theoretical explanation mentioned above, we can explain results for all *α*-amino acid esters in terms of specific solvation effects. A study of specific solvation effects on the *α*-amino acid esters showed that the log⁡_10_⁡*K* values show a decrease upon increase of the 1,4-dioxane ratio for all *α*-amino acid esters investigated. Because 1,4-dioxane should solvate B better than BH^+^, the log⁡_10_⁡*K* values, which are related to the formation of BH^+^, should decrease upon addition of 1,4-dioxane. We can say that the deviations from the trend with the log⁡⁡*β* values for all amino acid esters appeared in 1,4-dioxane-rich regions (*x* = 0.24), resulting from differences in solvent stabilization of the ionic species (H^+^ and BH^+^) being brought about by changing the percentage of 1,4-dioxane. The results show that each methyl ester is generally a slightly weaker base than its corresponding ethyl and t-butyl analogues. In addition, the alanine methyl ester is a weaker base than its benzyl ester. The acidity or basicity of a compound in a given medium is influenced by both the electronic effect of substituents and the solvent effect of the medium. Moreover, it is sometimes extremely difficult to assess how much each effect contributes to the acidity or basicity. In the literature it has also been shown that the methyl derivatives of aliphatic amines are weaker bases than their ethyl derivatives in water-methanol mixtures [49]. We can say that the basicity of a compound is a result of various factors such as

- the solvent effect (solvation power, the tendency of forming hydrogen bonds, selective solvation, dielectric constant, and the composition of the solution in the first solvation layer) in the case of mixed solvents,
- structural effects, electronic effects, and steric effects.

#### 3.3.3. Protonation Equilibria of DNA in Mixed Water-Dioxane Medium

The solvent effect on the acid dissociation constants of a ligand [87] can be summarized as follows.

- As the solvent dielectric constant decreases, the log⁡_10_⁡*K* of the ligand increases and vice versa.
- On decreasing the extent of hydrogen bonding in water by an organic solvent, the proton-accepting properties of the water increase, and consequently the log⁡_10_⁡*K* of the ligand decreases.
- Increasing proton solvation by an organic solvent is accompanied by a decrease in the log⁡_10_⁡*K* of ligand.

**Figure 10:**
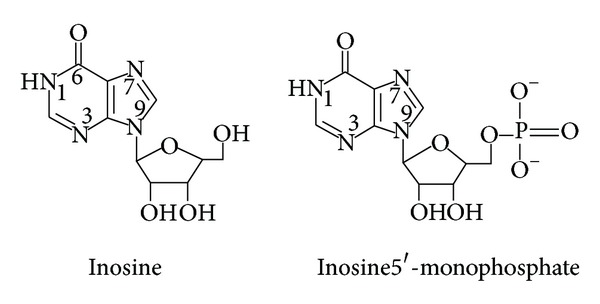


**Figure 11:**
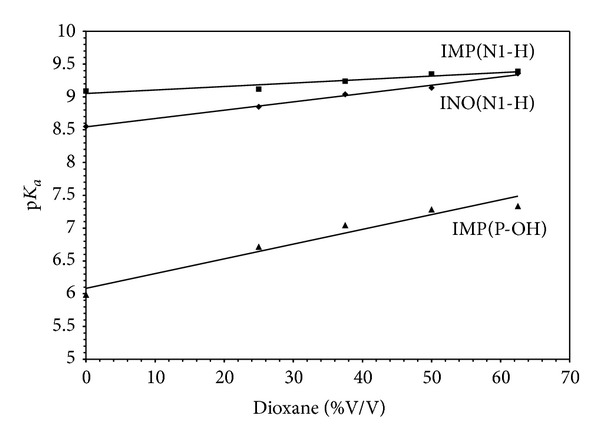
Effect of dioxane on the p*K*_a_ of inosine and inosine 5′-monophosphate (IMP).

The protonation constants of inosine and inosine 5′-monophosphate (Figure 10) as representative examples of DNA constituents in different compositions of dioxane-water mixtures reveal the following features (Table 10) [88]: p*K*_a_ (N1H) of inosine increases linearly with increasing percentage of organic solvent in the medium, Figure 11. This may be correlated with the ability of a solvent of relatively low dielectric constant to increase the electrostatic forces between the proton and the ligand anion and consequently the log⁡_10_⁡*K* value increases. The same trend was observed for the log⁡_10_⁡*K*'s of (N1H) and phosphoric acid (P-OH) groups of IMP, Figure 11. The log⁡_10_⁡*K* of the phosphate group in IMP is more affected by replacement of water molecules with dioxane. This is in agreement with the above finding, namely, the existence of hydrogen bonds between water molecules and phosphate ions. Thus, the phosphate ions are easily protonated in going from water to dioxane.

### 3.4. Protonation Equilibria in Mixed Water-DMSO Medium

The use of this mixed solvent has some advantages over pure DMSO. Thus, pure DMSO is very hygroscopic and controlling its water content is difficult [89]. This fact would affect reproducibility of experimental results. However, water-DMSO 50% : 50% mixture has only small hygroscopic character. A further advantage is its compatibility with the standard glass electrode, so that the pH measurements may be carried out in a similar way to that employed in a purely aqueous solution. In contrast, the use of pure DMSO is not recommended for potentiometry. Another advantage of the water-DMSO 50% : 50% mixture is its large acidity range (p*K*_w_ = 15.50) which allows the investigation of deprotonation equilibria of weak acids which could be hardly studied in water [90, 91]. Protonation constants of some *α*-amino acids at different water-DMSO mixtures and different temperatures are given in Tables 11 and 12 [92, 93].

#### 3.4.1. Protonation Equilibria of Amino Acids in Water-DMSO Medium

**Figure 12:**
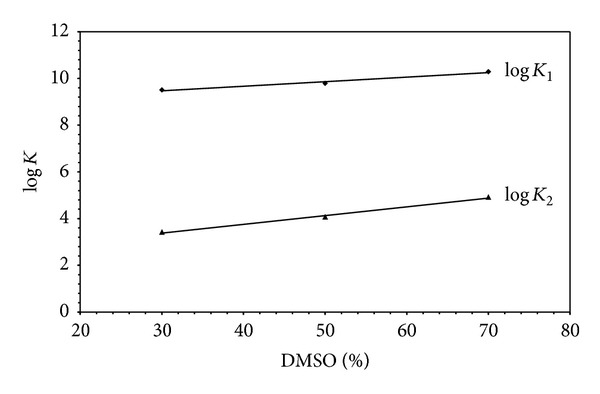
The variation of protonation constants of glycine with the percentage of DMSO.

The stoichiometric protonation constants of glycine, L-alanine, L-phenylalanine, L-threonine, and L-methionine in water-DMSO mixtures are given in Table 11. The protonation constants *K*_1_ and *K*_2_ are related to the protonation of the amino nitrogen and the carboxyl oxygen, respectively. When the change of log⁡_10_⁡*K*_1_ with the solvent composition, given in Table 11, is examined for *α*-amino acids, it is observed that these values increase with an increasing percent of dimethyl sulfoxide. The linear relation is given in Figure 12, for glycine as a representative example of *α*-amino acids. This can be explained by structural changes in the amino acids as the medium becomes more like dimethyl sulfoxide. The neutral form of the amino acids (HL) is subjected to a larger amount of solvation, compared to the anionic form (L^−^) in dimethyl sulfoxide rich media. In water-rich media, however, the reverse will be the case.

Also, the log⁡_10_⁡*K*_2_ values increase for each *α*-amino acid as the percentage of dimethyl sulfoxide in the solvent mixtures increases. Many studies have shown that the equilibrium constant is linearly related to the fraction of the organic solvent [80, 81, 83]. In general, the protonation of compounds containing O–H increases with increasing organic content of the solvent, due to the decrease in the dielectric constant of the bulk solvents. As the dielectric constant decreases, the ion-ion interaction involving the proton and the anionic oxygen donor of the ligand increases to a greater extent than the ion-dipole interaction between the proton and the solvent molecule. Thus the log⁡_10_⁡*K* of carboxylic acid (COO^−^H^+^) groups of *α*-amino acids increases linearly with increasing percentage of organic solvent in the medium. The same trend was observed for the p*K*_a_'s of phosphoric acid (P-OH) groups of IMP [88].

**Figure 13:**
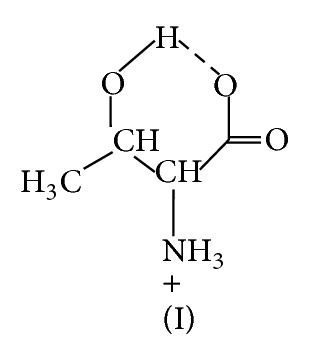
Intramolecular H-bond of serine.

Serine is *α*-amino acid containing three functional groups (–OH, –NH_2_, and COOH), but only two protonation equilibria are determined corresponding to the protonation of amino and carboxylate groups, respectively. This can be attributed to the fact that, as the relative pH in most experiments did not extend to pH > 12, the protonation constant for the aliphatic OH group in serine could not be measured. Also, a qualitative explanation could be based on the formation of an intramolecular hydrogen bond as depicted in Figure 13. Assume that the proton on this –OH group is more difficult to remove in the amino acid form.

Also, the log⁡_10_⁡*K*_2_ values increase for each *α*-amino acid as the percentage of dimethyl sulfoxide in the solvent mixtures increases. Many studies have shown that the equilibrium constant is linearly related to the fraction of the organic solvent [80, 81, 83].

### 3.5. Thermodynamic Quantities Associated with the Protonation Equilibria

Changes in the temperature may have some significant influences on the equilibrium properties and cause spontaneous changes in aquatic systems. Solution equilibria may be shifted to the right or to the left by an increase in temperature. The direction and magnitude of the shift are given by the sign and magnitude of the heat of the reaction as a function of temperature.

The thermodynamic parameters are useful tools for studying these interactions with bioligands and understanding the relative stability of the formed complexes.

The enthalpy changes accompanying complex formation can be obtained directly through calorimetric measurements, or indirectly from the temperature dependence of the stability constants via the Van't Hoff equation. The accuracy of Δ*H* values based on calorimetric measurements is generally higher than the accuracy of those based on the temperature dependence of log⁡_10_⁡*K* values. However, many parameters influence the accuracy of calorimetric data, for example, the enthalpy of dissociation of water, the heat of dilution, and so forth [93].

Application of the temperature dependence of log⁡_10_⁡*K* generally requires the determination of very accurate stability constants over as wide range in temperature as possible. However, the wider the range employed, the greater the uncertainty in the calculated values, because of the temperature dependence of the enthalpy values.

A high electrolyte concentration was used in order to keep variations of the activity coefficients at a minimum. Precise thermodynamic data can only be obtained provided that an inert electrolyte of fairly high concentration (≥0.1 mol*·*dm^−3^) is used. The main differences between different procedures for the study of ionic equilibria in aqueous and nonaqueous solutions are due to the activity coefficients. As in most equilibria in nonaqueous medium, a background electrolyte is added to maintain constant ionic strength ranging from about 0.1 to 0.3 mol*·*dm^−3^. This is allowed in some water-ethanol or water-dioxane mixtures, but not in solvents of low dielectric constants where the solubility of electrolyte is very low. In rigorous thermodynamic calculations, the equilibrium constant should be expressed in terms of activities of the component ions at equilibrium. However, for convenience, concentrations are generally used, where, for a particular species *i*, *a*_*i*_ = (*C*_*i*_)*F*_*i*_, where *C*_*i*_ is the concentration of the ion *i*, *a*_*i*_ is its activity, and *F*_*i*_ is its activity coefficient. The activity coefficients are maintained constant by working in a medium of constant ionic strength.

The results are summarized in Tables 12 and 13 and can be interpreted as follows.

**Figure 14:**
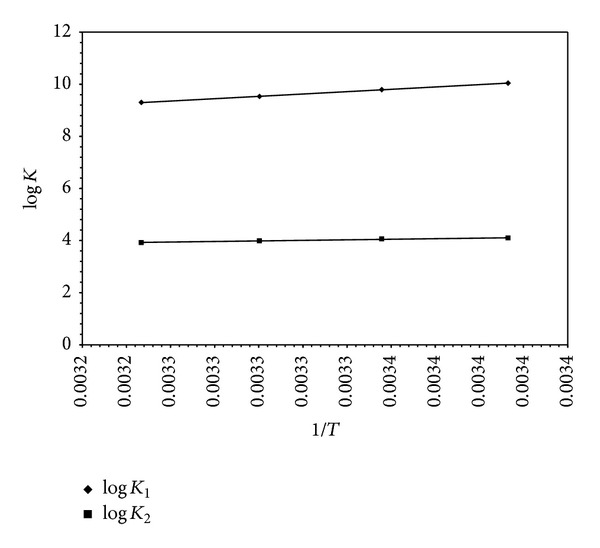
Van't Hoff plot of log⁡⁡*K* of glycine in 50% DMSO-50% H_2_O mixture (log⁡⁡*K*_1_ denotes protonation of amino group and log⁡⁡*K*_2_ denotes protonation of carboxylate group).

1. The protonation reactions (L^−^ + H^+^⇌LH^±^, corresponding to –NH_2_ group) and (LH^±^ + H^+^⇌LH_2_^+^, corresponding to carboxylate group) in Table 12 of the investigated amino acids are exothermic (Figure 14). Three factors affect these protonation reactions:
  - the neutralization reaction, which is an exothermic process,
  - desolvation of ions, which is an endothermic process,
  - the change in the configuration and the arrangement of the hydrogen bonds around the free and protonated ligands.
2. The log⁡_10_⁡*K* values decrease with increasing temperature revealing that the acidity increases with increasing temperature (Tables 12 and 13).

The protonation reactions (1) and (2) in Table 13 [88] of the N1 site of inosine and inosine 5′-monophosphate are exothermic.

The phosphate ion in IMP is more solvated than its protonated form. Consequently it contributes more to the endothermic process upon protonation and the net Δ*H*° is only 0.32 kJ mol^−1^. Also, the phosphate ions form ordered hydrogen bonds with water molecules, which is confirmed by the large entropy change Δ*S*° = 114.6 JK^−1^ mol^−1^. This contributes to a negative free energy change Δ*G*° = 33.8 kJ mol^−1^. The positive entropies due to the release of ordered water molecules and the breaking of hydrogen bonds were found by Kramer-Schnabel and Linder [73] to give positive Δ*H*°, large Δ*S*°, and net negative Δ*G*° for the protonation and complexation reactions of organic monophosphates and copper ions. The log⁡_10_⁡*K* values decrease with increasing temperature revealing that its acidity increases with increasing temperature.

## 4. Conclusions

Great efforts should be made to collect data with as high quality as possible. This is often a time-consuming tough job but there are no short cuts in this respect. We would like to stress the importance of the following points.

- The amino acids have special importance among the other chemical molecules because they are the foundation stones of living organisms. The determination of the protonation constants is of great importance for the elucidation of numerous biological compounds.
- The knowledge of the protonation constants for these bioligands is a prerequisite to an understanding of their mechanism of action in both chemical and biological processes. Thus, it is hoped that these data will be a significant contribution to workers carrying out mechanistic studies in biological media. However, a fundamental understanding of the effects that structural variations have on the properties of the amino acid is essential in order to interpret and predict the properties of proteins correctly and also in order to explain the behaviors of enzymes in living organisms.
- In addition, the protonation constants of various amino acids investigated in this study not only are used for quantitative purposes but also can be used for the evaluation of the solvent effect and structural behavior of the amino acids in water containing media.
- Also, the present study supports the idea that the solvent effect on the protonation constants for the amino acids can be utilized to predict whether the zwitterion or the neutral form is the predominant species and how the zwitterionic to neutral form ratio changes with the concentration of organic component in water-organic solvent mixtures.
- The protonation constants of some dipeptides were determined in water and water-ethanol mixtures. Generally, it was observed that the log⁡_10_⁡*K*_1_ values of dipeptides decrease and the log⁡_10_⁡*K*_2_ values increase with the increase in the mole fraction of ethanol. The chemical and biological activity of these substances would be expected to vary with the degree of ionization. Therefore, a good knowledge of the protonation constants of dipeptides in nonaqueous media is of considerable interest.
- Additionally, the protonation constant of amino acid esters will be helpful in the determination of the microscopic equilibrium constants of the corresponding amino acids due to knowledge of the microscopic equilibrium constants of some compounds being necessary for the calculation of the concentration of each ionized species at any pH, which is important for the complete understanding of the physicochemical behavior of such molecules.
- The basicity of a compound is a result of various factors such as (i) the solvent effect (solvation power, the tendency of forming hydrogen bonds, selective solvation, dielectric constant, and the composition of the solution in the first solvation layer) in the case of mixed solvents and (ii) structural effects, electronic effects, and steric effects.
- Equilibrium constants are directly related to the solvent composition.
- The search for the equilibrium model must be unambiguous. The search for the speciation scheme as a service to the reader should be based on computerized methods and visualized in the form of diagrams.
- The discussed data in this study will make an important contribution to the literature.

## Figures and Tables

**Table 1:** The autoprotolysis constants (*K*_ap_) obtained for various water-solvent mixtures.

Medium	p*K*_ap_
Water	13.78^a^, 13.69^b^, 13.77^c^
20% ethanol—80% water	14.03^d^
30% ethanol—70% water	14.17^a^, 14.16^b^
40% ethanol—60% water	14.30^d^
50% ethanol—50% water	14.40^a^, 14.28^b^
60% ethanol—40% water	14.48^d^
70% ethanol—30% water	14.67^a^, 14.59^b^
12.5% dioxane—87.5% water	14.17^e^
20% dioxane—80% water	14.31^f^, 14.22^b^
25% dioxane—75% water	14.37^e^
37.5% dioxane—62.5% water	14.50
40% dioxane—60% water	14.89^f^, 14.86^b^
50% dioxane—50% water	15.44^e^
60% dioxane—40% water	15.54^f^, 15.62^b^
62.5% dioxane—37.5% water	15.70^e^
70% dioxane—30% water	15.75^e^
50% DMSO—50% water	15.48^g^

^a^Data taken from reference [38], ^b^data taken from reference [37],^ c^data taken from reference [39], ^d^data taken from reference [40], ^e^data taken from reference [41], ^f^data taken from reference [42], and ^g^data taken from reference [43].

**Table 2:** The autoprotolysis constants (*K*_ap_) obtained for various water-organic solvent mixtures measured in an ionic medium of 0.1 mol*·*dm^−3^ NaClO_4_ at 25°C^a^.

Organic component	Organic component %								
	0	10	20	30	40	50	60	70	80
Methanol	13.69	13.75	13.78	13.70	13.73	13.71	13.72	13.77	13.94
Ethanol	13.69	13.82	13.99	14.16	14.24	14.28	14.39	14.59	14.77
1-Propanol	13.69	13.78	13.98	14.04	14.15	14.31	14.46	14.61	14.82
2-Propanol	13.69	13.76	13.95	14.05	14.26	14.34	14.56	14.79	—
Dioxane	13.69	13.91	14.22	14.51	14.86	15.24	15.62	—	—
Acetonitrile	13.69	13.96	14.19	14.44	14.74	15.04	—	—	—

^a^Data taken from reference [37].

The % is expressed as (volume/Volume).

**Table 3:** Protonation constants of some *α*-amino acids in water and water-ethanol mixtures [Temp. = 25°C and *I* = 0.1 mol*·*dm^−3^ NaCl].

AA	Water			30% E—70% W			50% E—50% W			70% E—30% W		
	log⁡*K*_1_	log⁡*K*_2_	log⁡*K*_3_	log⁡*K*_1_	log⁡*K*_2_	log⁡*K*_3_	log⁡*K*_1_	log⁡*K*_2_	log⁡*K*_3_	log⁡*K*_1_	log⁡*K*_2_	log⁡*K*_3_
Cys	10.29	8.36	1.71	10.25	8.23	2.60	10.81	7.06	2.93	12.07	6.33	2.82
Tyr	10.14	9.03	2.17	10.46	9.03	2.48	10.90	9.06	3.25	10.70	8.80	2.69
Trp	9.33	2.35	—	9.17	2.83	—	9.05	3.21	—	8.78	2.77	—
Lys	10.69	9.08	2.04	10.20	8.77	1.71	10.07	8.76	2.68	9.64	8.48	2.19
His	9.08	6.02	1.70	8.86	6.21	3.00	8.53	5.85	2.93	8.32	5.73	2.99

*Note.* E: ethanol, W: water, Cys = L-cysteine, Tyr = L-tyrosine, Trp = L-tryptophane, Lys = L-lysine, and His = L-histidine. ^a^Data taken from reference [50]; log⁡*K*_1_: corresponds to 11 species (i.e., L^−^ + H^+^⇌ LH); log⁡*K*_2_ corresponds to 12 species (i.e., LH + H^+^⇌LH_2_^+^). The % is expressed as (volume/Volume).

**Table 4:** Protonation constants of some *α*-amino acids in water and water-ethanol mixtures [Temp. = 25°C and *I* = 0.1 mol*·*dm^−3^ NaClO_4_]^a^.

AA	30% E—70% W		40% E—60% W		50% E—50% W		60% E—40% W		70% E—30% W	
	log⁡*K*_1_	log⁡*K*_2_	log⁡*K*_1_	log⁡*K*_2_	log⁡*K*_1_	log⁡*K*_2_	log⁡*K*_1_	log⁡*K*_2_	log⁡*K*_1_	log⁡*K*_2_
Gly	9.54	2.81	9.39	2.89	9.35	2.90	9.20	3.05	9.05	3.06
Ala	9.70	2.88	9.55	2.97	9.45	3.05	9.18	3.06	9.12	3.08
Val	9.50	2.85	9.41	2.95	9.30	3.15	9.07	3.16	9.02	3.17
Leu	9.60	2.87	9.40	3.01	9.30	3.06	9.07	3.17	9.05	3.25
Ile	9.55	2.92	9.47	2.98	9.31	3.15	9.20	3.17	9.10	3.24
Phe	9.12	2.75	8.95	2.95	8.79	2.98	8.70	3.05	8.60	3.00
Ser	9.18	2.88	9.00	2.87	8.77	2.89	8.68	2.91	8.66	2.90
Met	9.05	2.67	8.99	2.85	8.85	2.88	8.67	3.00	8.62	3.02

*Note*. E: ethanol, W: water, Val = L-valine, Ala = L-alanine, Leu = L-leucine, Ile = L-isoleucine, Phe = L-phenylalanine, Ser = L-serine, and Thr = L-threonine; ^a^data taken from reference [49]. log⁡*K*_1_ corresponds to 11 species (i.e., L^−^ + H^+^⇌ LH); log⁡*K*_2_ corresponds to 12 species (i.e., LH + H^+^⇌LH_2_^+^). The % is expressed as (volume/Volume).

**Table 5:** Protonation constants of dipeptides in water and water-ethanol mixture [Temp. = 25°C and *I* = 0.1 mol*·*dm^−3^ NaCl].

Dipeptides	Water		20% E—80% W^c^		40% E—60% W^c^		60% E—40% W^c^	
	log⁡*K*_1_	log⁡*K*_2_	log⁡*K*_1_	log⁡*K*_2_	log⁡*K*_1_	log⁡*K*_2_	log⁡*K*_1_	log⁡*K*_2_
Gly-Gly	8.15^a^	3.11^b^	7.66	3.33	6.92	3.54	6.98	3.81
GlyVal	8.23^d^	3.15^d^	7.67	3.52	7.06	3.91	6.92	4.18
GlyLeu	7.91^e^	3.15^e^	7.60	3.38	7.17	3.94	7.02	4.22
GlyPhe	7.98^d^	3.03^d^	7.62	3.33	6.96	3.65	6.98	3.91
GlyThr	8.50^c^	3.20^c^	7.74	3.19	7.07	3.37	7.05	3.67
GlyMet	8.22^f^	3.11^f^	7.64	3.32	7.02	3.62	6.96	3.91

*Note.* E: ethanol; W: water; ^a^data taken from reference [51]; ^b^data taken from reference [52]; ^c^data taken from reference [42]; ^d^data taken from reference [53]; ^e^data taken from reference [54]; ^f^data taken from reference [55]. log⁡*K*_1_ corresponds to 11 species (i.e., L^−^ + H^+^⇌ LH); log⁡*K*_2_ corresponds to 12 species (i.e., LH + H^+^⇌LH_2_^+^). The % is expressed as (volume/Volume).

**Table 6:** Protonation constants of *α*-amino acid esters in water-ethanol mixtures [Temp. = 25°C and *I* = 0.1 mol*·*dm^−3^ NaCl]^a^.

Esters	Water		30% E—70% W		50% E—50% W		70% E—30% W	
	log⁡*K*_1_	log⁡*K*_2_	log⁡*K*_1_	log⁡*K*_2_	log⁡*K*_1_	log⁡*K*_2_	log⁡*K*_1_	log⁡*K*_2_
CysOMe	9.17	6.38	9.23	6.09	9.34	5.88	10.82	6.24
CysOEt	9.36	6.54	9.37	6.17	9.48	5.97	11.13	6.28
TyrOMe	9.73	7.04	10.10	6.73	10.49	6.42	10.62	6.17
TyrOEt	9.71	7.05	10.13	6.75	10.52	6.45	10.63	6.19
TrpOMe	10.66	7.10	11.27	6.78	11.56	6.51	11.39	6.29
TrpOEt	10.79	7.10	11.28	6.98	11.70	6.80	11.77	6.45
LysOMe	9.99	6.98	9.80	6.90	9.52	6.63	9.12	6.46
LysOEt	10.32	7.18	9.70	6.81	9.45	6.60	9.00	6.35
HisOMe	7.10	4.96	6.72	4.78	6.54	4.61	6.34	4.39

*Note.* E = ethanol; W = water; ^a^data taken from reference [56]. log⁡*K*_1_ corresponds to 11 species (i.e., L^−^ + H^+^⇌ LH); log⁡*K*_2_ corresponds to 12 species (i.e., LH + H^+^⇌LH_2_^+^). The % is expressed as (volume/Volume).

**Table 7:** Protonation constants of *α*-amino acid-t-butyl and benzyl esters at 25°C for different water-EtOH mixtures [*I* = 0.1 mol*·*dm^−3^ NaCl]^a^.

Esters	20% E—80% W *X* = 0.0331	30% E—70% W *X* = 0.1167	40% E—60% W *X* = 0.1740	50% E—50% W *X* = 0.2356	60% E—40% W *X* = 0.3161	70% E—30% W *X* = 0.4183	80% E—0% W *X* = 0.5521
GlyO-t-Bu	7.66	7.51	7.40	7.30	7.20	7.05	6.64
AlaO-t-Bu	9.01	8.86	8.75	8.55	8.39	8.16	8.14
ValO-t-Bu	7.74	7.56	7.15	6.95	6.85	6.51	6.50
LeuO-t-Bu	7.79	7.63	7.38	7.21	7.02	6.92	6.75
PheO-t-Bu	7.15	7.00	6.67	6.56	6.50	6.40	6.33
IleO-t-Bu	7.75	7.65	7.35	7.18	7.12	6.89	6.64
GlyO-Bz	7.27	—	7.07	—	6.94	—	6.85
AlaO-Bz	7.35	—	7.17	—	7.01	—	7.00
ValO-Bz	7.16	—	6.88	—	6.77	—	6.68
SerO-Bz	6.75	—	6.60	—	6.51	—	6.54

*Note.* E = ethanol; W = water; ^ a^data taken from ref. [57]. log⁡*K*_1_ corresponds to 11 species (i.e., L^−^ + H^+^⇌ LH); log⁡*K*_2_ corresponds to 12 species (i.e., LH + H^+^⇌LH_2_^+^). The % is expressed as (volume/Volume).

**Table 8:** Protonation constants of some *α*-amino acids in water and water-dioxane mixtures [Temp. = 25°C and *I* = 0.1 mol*·*dm^−3^ NaClO_4_]^a^.

AA	Water		10% D—90% W		20% D—80% W		30% D—70% W		40% D—60% W		50% D—50% W		60% D—40% W	
	log⁡*K*_1_	log⁡*K*_2_	log⁡*K*_1_	log⁡*K*_2_	log⁡*K*_1_	log⁡*K*_2_	log⁡*K*_1_	log⁡*K*_2_	log⁡*K*_1_	log⁡*K*_2_	log⁡*K*_1_	log⁡*K*_2_	log⁡*K*_1_	log⁡*K*_2_
Gly	9.58	2.32	9.55	2.41	9.56	2.70	9.58	2.87	9.58	3.15	9.60	3.48	9.62	3.60
Ala	9.70	2.40	9.66	2.52	9.72	2.64	9.68	2.89	9.73	3.10	9.75	3.39	9.78	3.63
Val	9.61	2.38	9.56	2.53	9.60	2.68	9.58	2.86	9.60	3.08	9.62	3.43	9.66	3.65
Leu	9.75	2.50	9.59	2.64	9.64	2.80	9.66	2.99	9.70	3.31	9.72	3.55	9.75	3.75
Ile	9.76	2.47	9.72	2.58	9.63	2.74	9.67	2.97	9.65	3.26	9.70	3.55	9.71	3.74
Phe	9.20	2.43	9.15	2.57	9.10	2.64	9.12	2.81	9.15	3.10	9.16	3.36	9.15	3.50
Ser	9.15	2.42	9.07	2.57	9.10	2.62	9.13	2.82	9.16	3.04	9.20	3.17	9.17	3.35
Thr	9.04	2.45	9.02	2.53	9.06	2.60	9.05	2.75	9.07	2.96	9.10	3.18	9.17	3.40

*Note.* D = dioxane, W = water, Val = DL-valine, Ala = DL-alanine, Leu = L-leucine, Ile = L-isoleucine, Phe = DL-phenylalanine, Ser = L-serine, and Thr = L-threonine. ^a^Data taken from reference [61]. log⁡*K*_1_ corresponds to 11 species (i.e., L^−^ + H^+^⇌ LH); log⁡*K*_2_ corresponds to 12 species (i.e., LH + H^+^⇌LH_2_^+^). The % is expressed as (volume/Volume).

**Table 9:** Protonation constants of *α*-amino acid esters in water-1,4-dioxane mixtures [Temp. = 25°C and *I* = 0.1 mol*·*dm^−3^ NaCl]^a^.

Esters	*p*	*q* ^a^	Water	20% D—80% W (*X* = 0.05)	40% D—60% W (*X* = 0.12)	60% D—40% W (*X* = 0.24)
			log⁡K	log⁡K	log⁡K	log⁡K
GlyO-Me	1	1	7.67	7.47	7.18	6.85
GlyO-t-Bu	1	1	8.38	8.21	7.91	6.97
ValO-Me	1	1	7.53	7.38	7.09	6.08
ValO-Et	1	1	8.86	8.01	7.15	6.58
ValO-t-Bu	1	1	9.05	8.54	7.71	7.13
SerO-Me	1	1	7.10	6.99	6.69	6.61
SerO-Et	1	1	7.46	7.25	6.84	6.79
LeuO-Me	1	1	7.66	7.47	7.19	6.32
LeuO-Et	1	1	7.75	7.78	7.31	6.73
LeuO-t-Bu	1	1	7.89	8.04	7.60	6.95
PheO-Me	1	1	7.11	6.66	6.42	6.01
PheO-Et	1	1	8.12	7.24	6.81	6.24
PheO-t-Bu	1	1	8.26	7.39	6.96	6.64

*Note.* D = dioxane, W = water; ^a^data taken from ref. [44]. log⁡K_1_ corresponds to 11 species (i.e., L^−^ + H^+^⇌ LH).

**Table 10:** Protonation constants of inosine and IMP in dioxane-water media at 25°C and *I* = 0.1 mol*·*dm^−3^ NaCl.

System	% dioxane	*p*	*q* ^a^	log⁡*β* ^b^
Inosine IMP^c^	0%	1	1	8.55
		1	1	9.09
		1	2	15.07

Inosine IMP	25%	1	1	8.85
		1	1	9.12
		1	2	15.83

Inosine IMP	37.5%	1	1	9.04
		1	1	9.24
		1	2	16.28

Inosine IMP	50%	1	1	9.14
		1	1	9.35
		1	2	16.63

Inosine IMP	62.5%	1	1	9.36
		1	1	9.39
		1	2	16.72

^a^ *p* and *q* are stoichiometric coefficients corresponding to ligand and H^+^, respectively; ^b^data taken from reference [83], ^c^IMP = inosine 5′-monophosphate. The % is expressed as (volume/Volume). Species 11 refers to L^−^ + H^+^⇌ LH; species 12 refers to L + 2H^+^⇌LH_2_^+^.

**Table 11:** Protonation constants of some *α*-amino acids in water and DMSO-water media at 25°C and *I* = 0.1 mol*·*dm^−3^ NaNO_3_^a^.

System	*p*	*q* ^b^	30% DMSO	50% DMSO	70% DMSO
Gly	1	1	9.51 ± 0.01	9.79 ± 0.01	10.29 ± 0.01
	1	2	12.92 ± 0.01	13.85 ± 0.02	15.20 ± 0.01

L-Ala	1	1	9.65 ± 0.009	9.85 ± 0.02	10.48 ± 0.02
	1	2	13.07 ± 0.01	13.96 ± 0.03	15.36 ± 0.03

L-Val	1	1	9.34 ± 0.01	9.57 ± 0.01	9.99 ± 0.01
	1	2	12.61 ± 0.02	13.61 ± 0.02	14.88 ± 0.03

L-Ser	1	1	8.95 ± 0.01	9.12 ± 0.02	9.89 ± 0.01
	1	2	12.16 ± 0.02	12.95 ± 0.03	14.71 ± 0.03

L-Isl	1	1	9.31 ± 0.01	9.51 ± 0.02	9.91 ± 0.02
	1	2	12.75 ± 0.01	13.56 ± 0.03	15.02 ± 0.03

L-Leu	1	1	9.46 ± 0.01	9.77 ± 0.05	10.18 ± 0.01
	1	2	12.80 ± 0.02	13.81 ± 0.05	14.98 ± 0.02

L-Phe	1	1	9.06^d^	9.35^c^	—
	1	2	12.44	13.43	

L-Met	1	1	9.07^d^	9.48^c^	—
	1	2	12.69	13.48	

L-Thr	1	1	8.95^d^	9.32^c^	—
	1	2	12.30	13.38	

^a^Data taken from reference [87]; ^b^ *p* and *q* are stoichiometric coefficients corresponding to ligand and H^+^, respectively; ^c^data taken from reference [88]. Species 11 refers to L^−^ + H^+^⇌ LH; species 12 refers to L + 2H^+^⇌LH_2_^+^; the % is expressed as (volume/Volume).

**Table 12:** Protonation constants (log_10_⁡*β*_*q*,*r*_) of some *α*-amino acids in solution containing 50% DMSO at different temperatures and *I* = 0.1 mol*·*dm^−3^ NaNO_3_ and their thermodynamic parameters^b^.

System	*p*	*q* ^a^	20°C	25°C	30°C	35°C	Thermodynamics^d^		
							Δ*H*°	Δ*S*°	Δ*G*°
Gly	1	1	10.04	9.79	9.53	9.30	−85.71	−100.14	−55.87
	1	2	14.14	13.85	13.52	13.22	−21.04	6.87	−23.09

L-Ala	1	1	10.02	9.85	9.69	9.46	−63.51	−24.63	−56.17
	1	2	14.19	13.96	13.75	13.46	−19.34	13.80	−23.46

L-Val	1	1	9.79	9.57	9.49	9.26	−57.69	−9.63	−54.82
	1	2	13.89	13.61	13.47	13.17	−21.75	4.31	−23.03

L-Ser	1	1	9.42	9.12	8.82	8.52	−103.66	−173.88	−52.02
	1	2	13.30	12.95	12.60	12.25	−17.28	15.34	−21.85

L-Isl	1	1	9.72	9.51	9.39	9.20	−58.07	−12.28	−55.74
	1	2	13.81	13.56	13.42	13.19	−11.06	40.55	−23.01

L-Leu	1	1	10.09	9.77	9.45	9.13	−110.57	−184.0	−55.74
	1	2	14.18	13.81	13.42	13.04	−21.07	6.51	−32.01

^a^DMSO% (vol/vol), ^b^data taken from reference [87], ^c^ *q* and *r* are stoichiometric coefficients corresponding to ligand and H^+^ respectively; ^d^With respect to the thermodynamic parameters (Δ*H*°, Δ*S*° and Δ*G*°) these parameters referred to these reactions (L^−^ + H^+^⇌ LH^±^, corresponding to –NH_2_ group) and (LH^±^ + H^+^⇌LH_2_^+^, corresponding to carboxylate group); The % is expressed as (volume/Volume).

**Table 13:** Effect of temperature and thermodynamic parameters of protonation equilibria of inosine and IMP^a^.

System	*T* (°C)	*p*	*q* ^b^	log⁡*β*	Equilibrium	Δ*H*° (kJ mol^−1^)	Δ*S*° (JK^−1^ mol^−1^)	Δ*G*° (kJ mol^−1^)
Inosine	15	1	1	8.77	Inosine	−35.0	46.3	−48.8
IMP	15	1	1	9.33	(1) L^−^ + H^+^⇌ LH			
		1	2	15.28				
Inosine	20	1	1	8.67				
IMP	20	1	1	9.17				
		1	2	15.17				
Inosine	25	1	1	8.55	IMP	−33.0	64.6	−52.2
IMP	25	1	1	9.09	(2) L^3−^+ H^+^⇌ LH^2−^	0.32	114.6	−33.8
		1	2	15.07	(3) LH^2−^ + H^+^⇌LH_2_^−^			
Inosine	30	1	1	8.44				
IMP	30	1	1	8.95				
		1	2	14.99				

^a^ *p* and *q* are stoichiometric coefficients corresponding to ligand and H^+^, respectively; ^b^data taken from reference [83]; L denotes inosine and inosine 5′-monophosphate.

## Abbreviations

Ala: Alanine
Cys: Cysteine
D: Dioxane
E: Ethanol
DMSO: Dimethyl sulfoxide
Gly: Glycine
Gly-Gly: Glycylglycine
GlyLeu: Glycylleucine
GlyMet: Glycylmethionine
GlyPhe: Glycylphenylalanine
GlyThr: Glycylthreonine
GlyVal: Glycylvaline
His: Histidine
IMP: Inosine 5′-monophosphate
Lys: Lysine
Met: Methionine
Trp: Tryptophane
Tyr: Tyrosine.
